# Safety and Efficacy of Lucitanib Plus Toripalimab in Advanced Solid Tumors Refractory to Standard Therapies: An Open‐Label, Multicenter, Phase II Study

**DOI:** 10.1002/mco2.70672

**Published:** 2026-03-11

**Authors:** Ting Zhou, Haishuang Sun, Gang Chen, Guoping Zhang, Jinsheng Wu, Shenhong Qu, Yaqian Han, Desheng Hu, Yang Ling, Yulong Zheng, Jian Liu, Lizhu Lin, Yongsheng Li, Jianji Pan, Yanyan Liu, Cuiying Wang, Guohong Fu, Jian Feng, Jianhua Shi, Huiming Cai, Meng Li, Fugen Li, Yinbin Wang, Li Zhang, Yunpeng Yang

**Affiliations:** ^1^ Department of Medical Oncology, State Key Laboratory of Oncology in South China, Guangdong Key Laboratory of Nasopharyngeal Carcinoma Diagnosis and Therapy, Guangdong Provincial Clinical Research Center for Cancer Sun Yat‐sen University Cancer Center Guangzhou China; ^2^ Department of Medical Oncology Yuebei People's Hospital Shaoguan China; ^3^ Department of Radiation Oncology The First Affiliated Hospital of Hainan Medical University Haikou China; ^4^ Department of Otolaryngology Head and Neck The People's Hospital of Guangxi Zhuang Autonomous Region Nanning China; ^5^ The Second Department of Head and Neck Radiotherapy Hunan Cancer Hospital Changsha China; ^6^ Department of Radiotherapy Hubei Cancer Hospital Wuhan China; ^7^ Department of Medical Oncology Changzhou Cancer Hospital Changzhou China; ^8^ Department of Medical Oncology The First Affiliated Hospital, Zhejiang University School of Medicine Hangzhou China; ^9^ Phase I Clinical Trial Research Laboratory The First Affiliated Hospital, Zhejiang University School of Medicine Hangzhou China; ^10^ Department of Oncology The First Affiliated Hospital of Guangzhou University of Chinese Medicine Guangzhou China; ^11^ Department of Medical Oncology Chongqing University Cancer Hospital Chongqing China; ^12^ Department of Radiotherapy Fujian Cancer Hospital Fuzhou China; ^13^ Department of Lymphatic Integrative Medicine Henan Cancer Hospital Zhengzhou China; ^14^ Department of Medical Oncology Hainan Third People's Hospital Sanya China; ^15^ General Surgery Department Hainan Third People's Hospital Sanya China; ^16^ Department of Respiratory Medicine Affiliated Hospital of Nantong University Nantong China; ^17^ The Second Ward of the Department of Internal Medicine Linyi Cancer Hospital Linyi China; ^18^ Haihe Biopharma Co., Ltd Shanghai China

**Keywords:** efficacy, lucitanib, nasopharyngeal carcinoma, Phase II study, solid tumor, toripalimab

## Abstract

Lucitanib is a novel multi‐target inhibitor of vascular endothelial growth factor receptor 1–3, fibroblast growth factor receptor 1–3, and platelet‐derived growth factor receptor α/β. This open‐label, multicenter, single‐arm Phase II study evaluated lucitanib plus the anti‐programmed cell death 1 (PD‐1) antibody toripalimab in patients with advanced solid tumors refractory to standard therapies. Patients received lucitanib (10 mg) once daily plus toripalimab (240 mg) every 3 weeks until progression or unacceptable toxicity. The primary endpoint was investigator‐assessed objective response rate (ORR) and secondary endpoints included disease control rate, duration of response, progression‐free survival (PFS), overall survival, and safety. Among 131 patients across four cohorts (PD‐1–treated recurrent/metastatic nasopharyngeal carcinoma [NPC], PD‐1–naïve NPC, recurrent/metastatic endometrial cancer [EC], and other tumors), ORR was 34.1%, 45.8%, 38.5%, and 13.5%, respectively. Median PFS was 4.2 months (95% confidence interval [CI], 4.1–5.6), 6.5 months (95% CI, 4.0–not estimable [NE]), 5.6 months (95% CI, 2.78–11.21), and 9.7 months (95% CI, 5.4–NE). The most common Grade ≥ 3 treatment‐related adverse events were hypertension (37.4%), proteinuria (10.7%), and thrombocytopenia (10.7%). Lucitanib plus toripalimab showed encouraging antitumor activity with manageable safety in heavily pretreated advanced solid tumors, supporting further randomized evaluation, particularly in NPC and EC.

**Trial Registration**: Chinese Clinical Trial Registry Identifier: ChiCTR2400087935

## Graphical abstract

Lucitanib plus toripalimab showed manageable safety and encouraging antitumor activity in advanced solid tumors, particularly in PD‐1‐refractory nasopharyngeal carcinoma and recurrent endometrial carcinoma. Clinical responses were observed across subgroups regardless of prior immunotherapy.

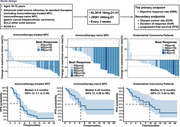

## 1 Introduction

Cancer remains a leading cause of morbidity and mortality worldwide [1]. Despite substantial advances in systemic therapy, patients with advanced or metastatic solid tumors who have progressed after standard treatments still face limited therapeutic options and poor outcomes. Therefore, there is an ongoing need to develop more effective and tolerable regimens for heavily pretreated populations.

Immune checkpoint inhibitors targeting the programmed cell death 1 (PD‐1)/programmed death‐ligand 1 (PD‐L1) axis have reshaped the treatment landscape across multiple malignancies [2, 3]. However, durable benefit is achieved only in a subset of patients, and both primary and acquired resistance are frequently observed, particularly in patients who have received multiple prior lines of therapy. Improving response rates and extending the durability of benefit from PD‐1 blockade remain major clinical challenges.

This unmet need is particularly evident in recurrent/metastatic nasopharyngeal carcinoma (R/M NPC) and advanced or recurrent endometrial cancer (EC). In pretreated R/M NPC, anti‐PD‐1 monotherapy yields a modest objective response rate (ORR) of 20%–34% [4, 5, 6]. In EC, clinically meaningful benefit from PD‐1 blockade is largely confined to microsatellite instability‐high (MSI‐high)/mismatch repair‐deficient (dMMR) tumors, which comprise only 13%–30% of cases [7, 8]. These limitations highlight the need for synergistic combination regimens to enhance and extend the efficacy of PD‐1‐based therapy in pretreated solid tumors.

Targeting tumor angiogenesis represents a promising strategy to augment immunotherapy [9]. Vascular endothelial growth factor (VEGF)‐driven abnormal vasculature promotes hypoxia, limits immune‐cell trafficking, and fosters an immunosuppressive tumor microenvironment; accordingly, anti‐angiogenic therapy may alleviate VEGF‐mediated immunosuppression and induce vascular normalization, thereby enhancing immune infiltration and improving the efficacy of PD‐1 blockade [10, 11]. Consistent with this rationale, accumulating clinical evidence indicates that combining immune checkpoint inhibitors with anti‐angiogenic agents can improve outcomes across multiple malignancies, including breast cancer [12], non‐small cell lung cancer [13], hepatocellular carcinoma [14], renal carcinoma [15], and so forth. Lucitanib (AL3810), an oral multi‐target tyrosine kinase inhibitor of vascular endothelial growth factor receptor (VEGFR) 1–3, fibroblast growth factor receptor (FGFR) 1–3, and platelet‐derived growth factor receptor (PDGFR) α/β, may concurrently suppress angiogenic signaling and reshape the immune microenvironment, providing a strong biological basis for its combination with PD‐1 inhibition [16].

Clinically, lucitanib has shown encouraging efficacy with a manageable safety profile in Phase II studies of advanced solid tumors [17, 18], and our Phase Ib study further suggested promising activity in heavily pretreated nasopharyngeal carcinoma (NPC) [19]. Toripalimab is a humanized immunoglobulin G4 (IgG4) monoclonal antibody against PD‐1 and has been approved by the National Medical Products Administration (NMPA) in China for the treatment of multiple solid tumors. However, despite the mechanistic rationale for combining anti‐angiogenic therapy with PD‐1 blockade, evidence for lucitanib plus toripalimab remains limited in heavily pretreated advanced solid tumors, particularly in EC and PD‐1 inhibitor‐resistant NPC. Therefore, we conducted this open‐label, multicenter, Phase II study to evaluate the efficacy and safety of lucitanib plus toripalimab in patients with advanced solid tumors receiving subsequent‐line treatment after progression on standard therapy, with a particular focus on clinically challenging subgroups, including EC and PD‐1 inhibitor‐resistant NPC.

## 2 Results

### 2.1 Patients

**FIGURE 1:**
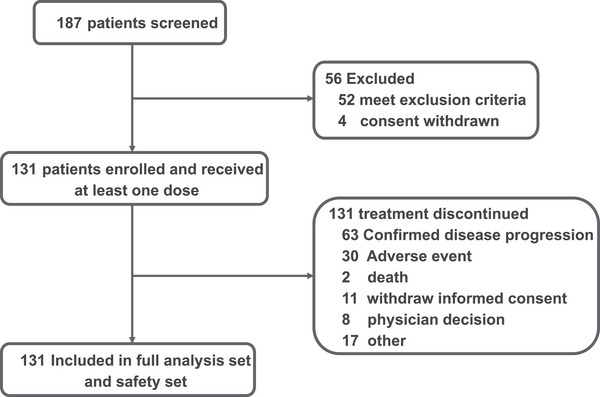
Study design and patient disposition. Flow diagram showing patient screening, enrollment, cohort allocation, treatment, and populations included in the efficacy and safety analyses.

Between May 11, 2021 and May 27, 2022, a total of 187 patients were screened, of whom 131 eligible patients were enrolled from 17 centers and received at least one dose of study treatment, and were therefore included in the efficacy and safety analyses (Figure 1). The enrolled population comprised immunotherapy‐treated NPC (*n* = 44, 33.6%), immunotherapy‐naïve NPC (*n* = 24, 18.4%), EC (*n* = 26, 19.8%), and other tumors (*n* = 37, 28.2%). The median age at enrollment was 54 years (range, 45–59), and most patients (*n* = 77, 58.8%) had an Eastern Cooperative Oncology Group (ECOG) performance status of 1. Prior radiotherapy had been administered to 68.7% of patients overall, including 88.6% and 95.8% of patients in the immunotherapy‐treated and immunotherapy‐naïve NPC cohorts, respectively, and 61.5% of those with EC. These data underscore the high burden of prior nasopharyngeal irradiation in the NPC cohorts, which is relevant to the risk of nasopharyngeal necrosis and hemorrhage observed with anti‐angiogenic therapy. Patients were heavily pretreated; approximately half had received more than two prior lines of systemic therapy for advanced disease, and in the immunotherapy‐treated NPC cohort, 33 of 44 patients (75%) had received two or more prior lines. Baseline demographic and disease characteristics by tumor cohort are summarized in Table 1.

**TABLE 1:** Baseline demographic and disease characteristics by tumor cohort.

Patient characteristics	All patients (*n* = 131)	NPC^a^ (*n* = 44)	NPC^b^ (*n* = 24)	EC (*n* = 26)	Others (*n* = 37)
Age, years, median (range)	54.0 (45.0–59.0)	47.5 (38.5–55.0)	51.0 (42.5–60.0)	56.5 (54.0–61.0)	56.0 (52.0–62.0)
BMI, mean ± SD	21.9 ± 3.1	21.0 ± 2.8	21.6 ± 2.8	23.3 ± 3.1	22.2 ± 3.2
Sex, *n* (%)					
Male	71 (54.2)	35 (79.5)	12 (50.0)	0	24 (64.9)
Female	60 (45.8)	9 (20.5)	12 (50.0)	26 (100.0)	13 (35.1)
ECOG PS, *n* (%)					
0	54 (41.2)	17 (38.6)	7 (29.2)	9 (34.6)	21 (56.8)
1	77 (58.8)	27 (61.4)	17 (70.8)	17 (65.4)	16 (43.2)
Smoking status, *n* (%)					
Never smoker	98 (74.8)	32 (72.7)	19 (79.2)	25 (96.2)	22 (59.5)
Current or former smoker	33 (25.2)	12 (27.3)	5 (20.8)	1 (3.8)	15 (40.5)
Prior radiotherapy, *n* (%)	90 (68.7)	39 (88.6)	23 (95.8)	16 (61.5)	12 (32.4)
Previous lines of therapy for advanced disease, *n* (%)					
1	61 (47.7)	11 (25.0)	16 (66.7)	17 (65.4)	24 (64.9)
2	25 (19.5)	12 (27.3)	5 (20.8)	2 (7.7)	6 (17.1)
≥ 3	45 (32.8)	21 (47.7)	3 (12.5)	7 (26.9)	7 (19.0)

*Note*: Others: other advanced solid tumors, including hepatocellular carcinoma, gastric carcinoma, small cell lung cancer, and other less common tumor types.

Abbreviations: BMI, body mass index; ECOG, Eastern Cooperative Oncology Group; PS, performance status; SD, standard deviation.

^a^ Immunotherapy‐treated (immunotherapy‐refractory) nasopharyngeal carcinoma (NPC) cohort.

^b^Immunotherapy‐naïve (chemotherapy‐refractory) NPC cohort.

**FIGURE 2:**
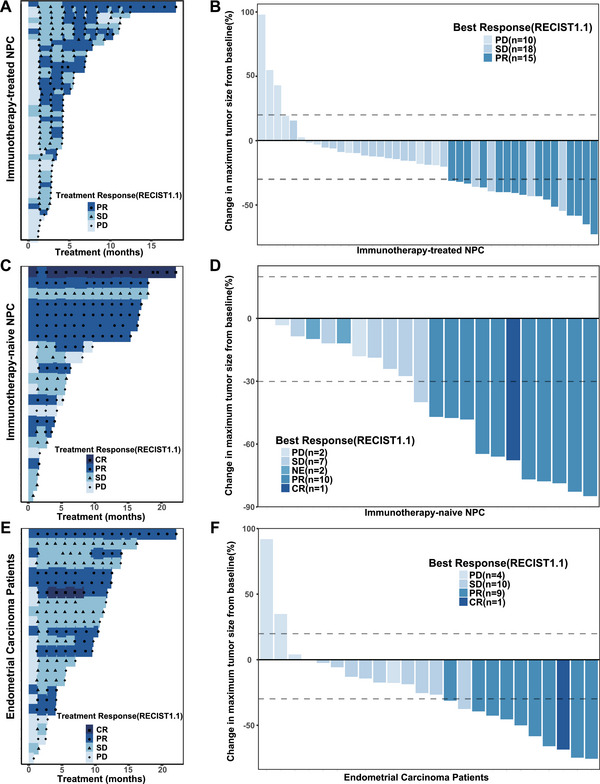
Tumor response and treatment exposure across tumor cohorts. (A) Swimmer plot showing treatment duration and time to tumor response in immunotherapy‐treated NPC patients. (B) Waterfall plot showing the best percentage change from baseline in target lesion size in immunotherapy‐treated NPC patients. (C) Swimmer plot showing treatment duration and time to tumor response in immunotherapy‐naïve NPC patients. (D) Waterfall plot showing the best percentage change from baseline in target lesion size in immunotherapy‐naïve NPC patients. (E) Swimmer plot showing treatment duration and time to tumor response in patients with EC. (F) Waterfall plot showing the best percentage change from baseline in target lesion size in EC patients. CR, complete response; EC, endometrial cancer; NE, not estimable; NPC, nasopharyngeal carcinoma; PD, progressive disease; PR, partial response; SD, stable disease.

At the data cut‐off on March 27, 2023, the median follow‐up duration was 17.02 months (range, 16.46–18.30). Median follow‐up times were similar across tumor cohorts, being approximately 15.87 months in the immunotherapy‐treated NPC cohort, 17.51 months in the immunotherapy‐naïve NPC cohort, 14.62 months in the EC cohort, and 21.52 months in the “other tumors” cohort (Table 2). All patients (100.0%) discontinued the study treatment regimen, with 63 (48.1%) experiencing disease progression and 30 (22.9%) encountering unacceptable toxicity. The duration of treatment for the immunotherapy‐treated NPC, immunotherapy‐naive NPC, EC patients, and other solid tumor patients are illustrated by swimmer plots in Figure 2A,C,E, respectively, while treatment exposure for patients with other solid tumors is shown in Figure S1A. These plots demonstrate that a substantial proportion of responding patients across cohorts received prolonged exposure to lucitanib plus toripalimab.

**TABLE 2:** Antitumor activity assessed by RECIST version 1.1.

	All patients (*n* = 131)	NPC^a^ (*n* = 44)	NPC^b^ (*n* = 24)	EC (*n* = 26)	Others (*n* = 37)
**Follow‐up duration, median (range)**	17.02 (16.46–18.30)	15.87 (12.58–17.02)	17.51 (16.56–18.53)	14.62 (11.99–16.95)	21.52 (20.67–21.82)
**Best overall response, no. (%)**					
CR	2 (1.5)	0	1 (4.2)	1 (3.8)	0
PR	39 (29.8)	15 (34.1)	10 (41.7)	9 (34.6)	5 (13.5)
SD	54 (41.2)	18 (40.9)	7 (29.2)	10 (38.5)	19 (51.4)
PD	25 (19.1)	10 (22.7)	2 (8.3)	4 (15.4)	9 (24.3)
NE	11 (8.4)	1 (2.3)	4 (16.6)	2 (7.7)	4 (10.8)
**ORR, no. (%)**	41 (31.3)	15 (34.1)	11 (45.8)	10 (38.5)	5 (13.5)
95% CI	23.5–40.0	20.5–49.9	25.6–67.2	20.2–59.4	4.50–28.88
**DCR, no. (%)**	95 (72.5)	33 (75.0)	18 (75.0)	20 (76.9)	24 (64.9)
95% CI	64.0–80.0	59.7–86.8	53.3–90.2	56.4–91.0	47.5–79.8

*Note*: Others: other advanced solid tumors, including hepatocellular carcinoma, gastric carcinoma, small cell lung cancer, and other less common tumor types.

Abbreviations: CI, confidence interval; CR, complete response; DCR, disease control rate; EC, endometrial cancer; NE, not evaluable; NPC, nasopharyngeal carcinoma; ORR, objective response rate; PD, progressive disease; PR, partial response; SD, stable disease.

^a^ Immunotherapy‐treated (immunotherapy‐refractory) NPC cohort.

^b^Immunotherapy‐naïve (chemotherapy‐refractory) NPC cohort.

### 2.2 Antitumor Activity

For immunotherapy‐treated NPC patients, the trial successfully completed both the first and second stages of the Simon's two‐stage design, and the null hypothesis of an ORR of 20% was rejected. At the time of data analysis, 15 of 44 patients achieved confirmed PR, yielding an ORR of 34.1% (95% confidence interval [CI], 20.5–49.9), while an additional 18 patients achieved stable disease (SD), resulting in a disease control rate (DCR) of 75% (95% CI, 59.7–86.8) (Table 2). Consistent with these response outcomes, tumor shrinkage in target lesions was observed in the majority of patients (*n* = 37, 84.1%) (Figure 2B).

In the immunotherapy‐naïve NPC cohort, objective responses were observed in 11 of 24 patients, including one CR and 10 PRs, corresponding to an ORR of 45.8% (95% CI, 25.6–67.2). Seven additional patients achieved SD, yielding a DCR of 75% (95% CI, 53.3–90.2) (Table 2). Notably, all patients in this cohort exhibited tumor shrinkage in target lesions (Figure 2D).

In the EC subgroup, 10 of 26 patients achieved an objective response, resulting in an ORR of 38.5% (95% CI, 20.2–59.4), while another 10 patients experienced SD, corresponding to a DCR of 76.9% (95% CI, 56.4–91.0) (Table 2). Tumor response dynamics in EC patients are illustrated in Figure 2F. Among patients with other tumor types, including hepatocellular carcinoma, gastric carcinoma, and small cell lung cancer, the ORR was 13.5% (95% CI, 4.5–28.8) (Table 2), with tumor response patterns summarized in Figure S1B.

**FIGURE 3:**
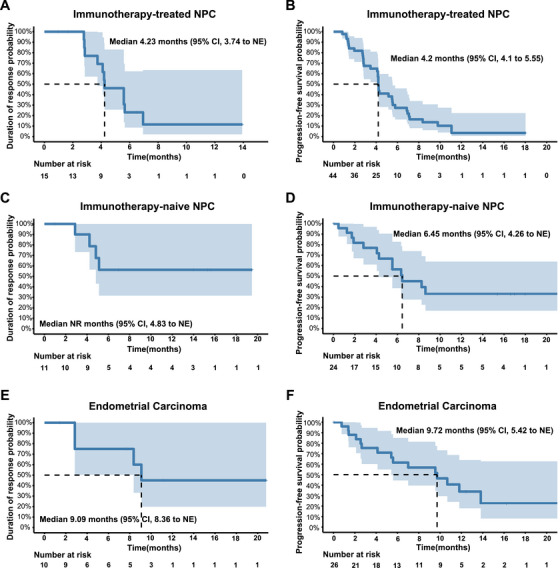
Duration of response and progression‐free survival. (A) Kaplan–Meier curve of DoR in responding patients with immunotherapy‐treated NPC. (B) Kaplan–Meier curve of PFS in the intention‐to‐treat population with immunotherapy‐treated NPC. (C) Kaplan–Meier curve of DoR in responding patients with immunotherapy‐naïve NPC. (D) Kaplan–Meier curve of PFS in the intention‐to‐treat population with immunotherapy‐naïve NPC. (E) Kaplan–Meier curve of DoR in responding patients with EC. (F) Kaplan–Meier curve of PFS in the intention‐to‐treat population with EC. DoR, duration of response; EC, endometrial cancer; NE, not estimable; NPC, nasopharyngeal carcinoma; PFS, progression‐free survival.

Among responders, the median duration of response (DoR) in immunotherapy‐treated NPC patients was 4.23 months (95% CI, 3.74–not estimable [NE]) (Figure 3A). In the overall immunotherapy‐treated NPC cohort, the median progression‐free survival (PFS) was 4.2 months (95% CI, 4.10–5.55) (Figure 3B), with a 6‐month PFS rate of 27.35% (95% CI, 14.46–41.94). In the immunotherapy‐naïve NPC cohort, the median DoR had not been reached at the time of analysis (Figure 3C), and 13 patients experienced disease progression or death events, resulting in a median PFS of 6.45 months (95% CI, 4.26‐–NE) (Figure 3D). The PFS rates at 6 and 12 months were 56.38% (95% CI, 32.67–74.57) and 32.89% (95% CI, 13.28–54.20), respectively.

In the EC subgroup, the median DoR among responding patients was 9.09 months (95% CI, 8.36–NE) (Figure 3E). A total of 15 PFS events were observed, and the median PFS was 9.72 months (95% CI, 5.42–NE) (Figure 3F). Among patients with other tumor types, the median DoR was not reached (Figure S2A), and the median PFS was 5.55 months (95% CI, 2.78–11.21) (Figure S2B), with a 12‐month PFS rate of 21.38% (95% CI, 8.41–38.24).

**FIGURE 4:**
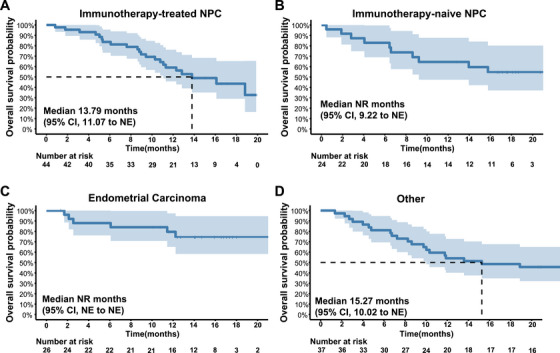
Overall survival by tumor cohort. (A) Kaplan–Meier curve of OS in patients with immunotherapy‐treated NPC. (B) Kaplan–Meier curve of OS in patients with immunotherapy‐naïve NPC. (C) Kaplan–Meier curve of OS in patients with EC. (D) Kaplan–Meier curve of OS in patients with other advanced solid tumors. EC, endometrial cancer; NE, not estimable; NPC, nasopharyngeal carcinoma; OS, overall survival.

At the time of analysis, 22 deaths had occurred in the immunotherapy‐treated NPC cohort, with a median OS of 13.79 months (95% CI, 11.07–NE) (Figure 4A). In the immunotherapy‐naïve NPC cohort, the median OS had not yet been reached (Figure 4B). Similarly, the median OS was not reached in the EC subgroup (Figure 4C). Among patients with other tumor types, the median OS was 15.27 months (95% CI, 10.02–NE) (Figure 4D).

Given that ORR was the primary endpoint of this Phase II study, OS analyses are secondary and descriptive; OS follow‐up is still ongoing, and the current OS estimates, particularly in cohorts where the median OS has not yet been reached, should therefore be interpreted with appropriate caution.

### 2.3 Safety

All patients were included in the safety analysis. Among them, treatment‐related adverse events (TRAEs) of any grade occurred in 130 out of 131 patients (99.2%), with Grade ≥ 3 TRAEs observed in 88 patients (67.2%) (Table 3). The most common Grade 3–4 TRAEs were hypertension (*n* = 49, 37.4%), proteinuria (*n* = 14, 10.7%), and thrombocytopenia (*n* = 14, 10.7%) (Table 3). Additionally, 33 (25.2%) experienced treatment‐related serious adverse events (SAEs). Hypothyroidism was the most frequent immune‐related adverse event (irAE), with the majority being Grade 1–2 (*n* = 78, 59.5%) (Table 3). Four had TRAE leading to death: three occurred in patients with NPC and were attributed to epistaxis, pharyngeal hemorrhage, and another cause, respectively; one in a non‐NPC tumor type was attributed to immune‐related pneumonitis (Table 3).

**TABLE 3:** Summary of safety results.

	All patients (*n* = 131)		NPC^a^ (*n* = 44)		NPC^b^ (*n* = 24)		EC (*n* = 26)		Others (*n* = 37)	
Patient characteristics	**Any grade**	**Grade** ≥ **3**	**Any grade**	**Grade** ≥ **3**	**Any grade**	**Grade** ≥ **3**	**Any grade**	**Grade** ≥ **3**	**Any grade**	**Grade** ≥ **3**
Any TRAE	43 (97.7)	88 (67.2)	24 (100.0)	26 (59.1)	26 (100.0)	18 (75.0)	37 (100.0)	22 (84.6)	130 (99.2)	22 (59.5)
SAE	33 (25.2)		9 (20.5)		6 (25.0)		8 (30.8)		10 (27.0)	
irAE	101 (77.1)	14 (10.7)	27 (61.4)	4 (9.1)	19 (79.2)	2 (8.3)	22 (84.6)	3 (11.5)	33 (89.2)	5 (13.5)
TRAEs occurring in ≥ 10% patients										
Hypertension	98 (74.8)	49 (37.4)	30 (68.2)	12 (27.3)	21 (87.5)	8 (33.3)	18 (69.2)	13 (50.0)	29 (78.4)	16 (43.2)
Proteinuria	93 (71.0)	14 (10.7)	27 (61.4)	8 (18.2)	15 (62.5)	3 (12.5)	22 (84.6)	2 (7.7)	29 (78.4)	1 (2.7)
Hypothyroidism	82 (62.6)	1 (< 1.0)	24 (54.5)	0	16 (66.7)	1 (4.2)	19 (73.1)	0	23 (62.2)	0
Thrombocytopenia	44 (33.6)	14 (10.7)	16 (36.4)	4 (9.1)	5 (20.8)	3 (12.5)	13 (50.0)	5 (19.2)	10 (27.0)	1 (2.7)
Leukopenia	43 (32.8)	2 (1.5)	13 (29.5)	1 (2.3)	7 (29.2)	0	14 (53.8)	0	9 (24.3)	1 (2.7)
Fatigue	38 (29.0)	4 (3.1)	8 (18.2)	0	7 (29.2)	0	11 (42.3)	1 (3.8)	12 (32.4)	3 (8.1)
Weight decreased	32 (24.4)	2 (1.5)	10 (22.7)	1 (2.3)	4 (16.7)	0	12 (46.2)	1 (3.8)	6 (16.2)	0
AST increased	32 (24.4)	2 (1.5)	5 (11.4)	0	8 (33.3)	0	9 (34.6)	1 (3.8)	10 (27.0)	1 (2.7)
Hypoalbuminemia	30 (22.9)	0	7 (15.9)	0	3 (12.5)	0	10 (38.5)	0	10 (27.0)	0
Neutropenia	29 (22.1)	2 (1.5)	8 (18.2)	1 (2.3)	4 (16.7)	1 (4.2)	11 (42.3)	0	6 (16.2)	0
ALT increased	26 (19.8)	2 (1.5)	5 (11.4)	0	5 (20.8)	1 (4.2)	6 (23.1)	1 (3.8)	10 (27.0)	0
Hyperthyroidism	26 (19.8)	0	1 (2.3)	0	3 (12.5)	0	13 (50.0)	0	9 (24.3)	0
Hematuria	26 (19.8)	0	5 (11.4)	0	5 (20.8)	0	7 (26.9)	0	9 (24.3)	0
Hand‐foot syndrome	26 (19.8)	1 (< 1.0)	11 (25.0)	0	1 (4.2)	0	5 (19.2)	1 (3.8)	9 (24.3)	0
Diarrhea	25 (19.1)	3 (2.3)	7 (15.9)	1 (2.3)	3 (12.5)	0	10 (38.5)	2 (7.7)	5 (13.5)	0
Blood triglycerides increased	22 (16.8)	4 (3.1)	2 (4.5)	0	2 (8.3)	0	11 (42.3)	2 (7.7)	7 (18.9)	2 (5.4)
Anemia	20 (15.3)	5 (3.8)	8 (18.2)	2 (4.5)	2 (8.3)	2 (8.3)	6 (23.1)	1 (3.8)	4 (10.8)	0
Anorexia	19 (14.5)	0	9 (20.5)	0	2 (8.3)	0	2 (7.7)	0	6 (16.2)	0
Blood creatinine increased	19 (14.5)	0	7 (15.9)	0	1 (4.2)	0	6 (23.1)	0	5 (13.5)	0
Dysphonia	18 (13.7)	0	6 (13.6)	0	2 (8.3)	0	5 (19.2)	0	5 (13.5)	0
Rash	15 (11.5)	2 (1.5)	4 (9.1)	1 (2.3)	4 (16.7)	0	1 (3.8)	0	6 (16.2)	1 (2.7)
Epistaxis	14 (10.7)	1 (< 1.0)	11 (25.0)	1 (2.3)	3 (12.5)	0	0	0	0	0
Hypokalemia	14 (10.7)	2 (1.5)	4 (9.1)	0	1 (4.2)	1 (4.2)	1 (3.8)	0	8 (21.6)	1 (2.7)
Nausea	13 (9.9)	0	4 (9.1)	0	1 (4.2)	0	5 (19.2)	0	3 (8.1)	0
Blood cholesterol increased	13 (9.9)	0	4 (9.1)		1 (4.2)		3 (11.5)		5 (13.5)	
Hyperuricemia	13 (9.9)	0	2 (4.5)	0	0	0	8 (30.8)	0	3 (8.1)	0
Vomiting	13 (9.9)	0	5 (11.4)	0	1 (4.2)	0	3 (11.5)	0	4 (10.8)	0
TEAEs leading to death	10 (7.6)		2 (4.5)		5 (20.8)		3 (8.1)		0	
TRAEs leading to death	4 (3.1)		2 (4.5)		1 (4.2)		0		1 (2.7)	
TEAEs leading to dose reduction	55 (42.0)		20 (45.5)		10 (41.7)		15 (57.7)		10 (27.0)	
TEAEs leading to dose interruption of lucitanib	85 (64.9)		26 (59.1)		13 (54.2)		19 (73.1)		27 (73.0)	
TEAEs leading to permanent discontinuation of toripalimab	15 (11.5)		2 (4.5)		5 (20.8)		2 (7.7)		6 (16.2)	

*Note*: Others: other advanced solid tumors, including hepatocellular carcinoma, gastric carcinoma, small cell lung cancer, and other less common tumor types.

Abbreviations: ALT, alanine aminotransferase; AST, aspartate aminotransferase; EC, endometrial cancer; irAE, immune‐related adverse event; NPC, nasopharyngeal carcinoma; SAE, serious adverse event; TEAE, treatment‐emergent adverse event; TRAE, treatment‐related adverse event.

^a^ Immunotherapy‐treated (immunotherapy‐refractory) NPC cohort.

^b^Immunotherapy‐naïve (chemotherapy‐refractory) NPC cohort.

In prespecified subgroup analyses by tumor cohort, the overall incidence of TRAEs and Grade ≥ 3 TRAEs was high across all cohorts, but certain toxicity patterns differed between NPC and non‐NPC populations (Table 3). Grade ≥ 3 hypertension occurred in 49/131 (37.4%) patients overall, with rates of 20/68 (29.4%) in the combined NPC cohorts and 29/63 (46.0%) in the non‐NPC cohorts (EC and other tumors). Hypertension was generally manageable with standard antihypertensive therapy plus lucitanib dose interruption or reduction as per protocol, and only a minority of patients required permanent treatment discontinuation due to hypertension. In contrast, bleeding‐related TRAEs were largely confined to NPC; epistaxis occurred in 14/68 (20.6%) NPC patients versus 0/63 non‐NPC patients, and all three hemorrhagic TRAEs leading to death were observed in the NPC cohorts. Nasopharyngeal necrosis was documented in 3/68 (4.4%) NPC patients (one in the PD‐1 inhibitor‐resistant cohort and two in the chemotherapy‐resistant cohort), confirmed by magnetic resonance imaging and nasopharyngoscopy; two of these cases were Grade 4, and one patient experienced Grade 5 massive hemorrhage. No nasopharyngeal necrosis was observed in non‐NPC tumors, consistent with the higher baseline bleeding risk in previously irradiated nasopharyngeal primaries.

Eighty‐five patients (64.9%) required one or more dose interruptions for lucitanib due to adverse events (AEs). The reasons for lucitanib dose interruptions included thrombocytopenia (*n* = 19, 14.5%), proteinuria (*n* = 14, 10.7%), hypertension (*n* = 9, 6.9%), and hand‐foot syndrome (*n* = 8, 6.1%). Fifty‐five patients (42.0%) underwent dose reductions for lucitanib, primarily due to proteinuria (*n* = 15, 11.5%), thrombocytopenia (*n* = 9, 6.9%), and hypertension (*n* = 9, 6.9%). Fifteen patients (11.5%) discontinued toripalimab. The most common irAEs leading to toripalimab discontinuation were liver dysfunction (*n* = 6, 4.6%), pneumonia (*n* = 3, 2.3%), and immune myocarditis (*n* = 2, 1.5%) (Table 3).

## 3 Discussion

In this Phase II study, we assessed the efficacy and safety of combining an anti‐PD‐1 antibody with an anti‐angiogenesis inhibitor in previously heavily treated solid tumors. The combination of lucitanib and toripalimab demonstrated antitumor activity with a manageable safety profile. The most pronounced benefit was observed in pretreated NPC and recurrent EC, in which ORRs were 34.1% and 38.5% and median DoRs were 4.2 and 9.0 months, respectively. These results suggest that lucitanib plus toripalimab may represent a potential later‐line treatment option for selected patients with NPC or EC who have exhausted standard therapies, whereas findings in other tumor types should be regarded as exploratory.

The efficacy of lucitanib plus toripalimab was explored in multiple cancer types in our study, objective response was observed in 41 patients, with ORR of 31.3%. For patients with R/M NPC who are refractory to first‐line therapy, effective treatment options remain limited. Previous Phase II studies have shown moderate antitumor efficacy of anti‐PD‐1 antibody monotherapy in platinum‐resistant R/M NPC patients, with ORRs ranging from 20% to 34% [4, 6, 20, 21, 22]. Moreover, according to the results of KEYNOTE 122 study, anti‐PD‐1 monotherapy does not provide better survival benefit than chemotherapy in later‐line therapy [23]. Similarly, previous studies indicated that anti‐VEGF/VEGFR monotherapy show a comparable ORR of 20%–31.4% for patients with R/M NPC patients [24, 25, 26]. When placed in the context of these trials, our study adds to the evolving treatment landscape by showing that, in the PD‐1–naïve NPC cohort, the combination of lucitanib with an anti‐PD‐1 antibody achieved an ORR of 45.8% and a DCR of 75%, even in patients who had received two or more prior lines of antitumor therapy. Although cross‐trial comparisons should be interpreted with caution, these results suggest that this regimen may offer clinically meaningful activity in this setting.

From a mechanistic perspective, lucitanib is a multi‐target TKI that inhibits VEGFR, FGFR, and PDGFR, thereby blocking aberrant angiogenesis, promoting vascular normalization and potentially alleviating VEGF‐driven immunosuppression, which may enhance T‐cell trafficking and synergize with PD‐1 blockade. Based on this mechanistic rationale, future prospective trials with dedicated translational components are needed to evaluate candidate predictive biomarkers, including PD‐L1 expression, MSI/TMB status, angiogenesis‐related markers, immune gene signatures, and tumor‐infiltrating immune cells.

Recent studies have also shown encouraging activity of anti‐angiogenic agents combined with anti‐PD‐1 antibodies in R/M NPC [27, 28, 29]. However, patients who had previously received immunotherapy were often excluded from these trials. In our study, lucitanib plus toripalimab demonstrated antitumor activity even in PD‐1‐refractory patients, with an ORR of 34.1% and a median PFS of 4.2 months. Although these results should be interpreted with caution given the single‐arm design and limited sample size, they suggest that the combination of lucitanib and toripalimab may represent a potentially useful treatment option for patients who are resistant to front‐line anti–PD‐1–based therapy.

Immunotherapy with checkpoint inhibitors has revolutionized the treatment landscape for EC [30, 31, 32, 33, 34, 35]. Our study highlighted the promising efficacy of lucitanib plus toripalimab for patients with pretreated recurrent EC. The ORR for the combination therapy in unselected patients with EC was 38.5% (10 out of 26), and the median PFS was 9.72 months. Furthermore, the median DoR was 9.09 months (95% CI 8.36–not reached), which appears numerically comparable to or higher than outcomes reported in previous studies such as the pembrolizumab–lenvatinib combination (ORR 32%, median PFS 6.6 months) and the nivolumab–cabozantinib combination (ORR 25%, median PFS 5.3 months); however, these cross‐trial comparisons are descriptive and limited by differences in study design and patient selection and should not be interpreted as evidence of superiority [36, 37].

Taken together, the most notable clinical benefits of lucitanib plus toripalimab were seen in advanced NPC, including immunotherapy‐resistant cases, and in EC. These findings highlight its potential as a later‐line option for two difficult‐to‐treat populations: PD‐1‐refractory NPC and EC. In contrast, responses in gastric cancer, hepatocellular carcinoma, and small cell lung cancer were limited, though firm conclusions are precluded by the small sample sizes, warranting further investigation.

The safety profiles of lucitanib and toripalimab observed in this study were consistent with previous studies. The combination of lucitanib and toripalimab was generally well tolerated, with a 67.2% incidence rate of Grade ≥ 3 TRAEs, and no evidence of new or late‐onset toxicity during the study. Hypertension (74.8%) was the most common TRAE, with Grade ≥ 3 hypertension occurring in 37.4% of patients overall and at numerically higher rates in non‐NPC than NPC cohorts, manageable through lucitanib dose adjustment and/or treating with antihypertensive drugs, aligning with previous reports for other anti‐angiogenic agents. Hypothyroidism (58%) was the most frequent irAE attributed to toripalimab, consistent with observations for other PD‐1 inhibitors [38].

Hand‐foot syndrome, a common adverse effect of small‐molecule VEGFR inhibitors [39], occurred at a lower frequency in our study (19.8%) compared to other anti‐angiogenic inhibitors, with only one patient experiencing Grade 3 hand‐foot syndrome. However, incidences of headache, epistaxis, and pharyngolaryngeal pain were notably higher in patients with R/M NPC compared to other solid tumor cohorts, likely attributable to the location of the primary tumor lesion and prior radiotherapy. Consistently, bleeding‐related TRAEs (including epistaxis and pharyngeal hemorrhage) and all hemorrhagic TRAEs leading to death occurred exclusively in the NPC cohorts, whereas no excess of local catastrophic hemorrhage was observed in non‐NPC tumors, highlighting the particular vulnerability of previously irradiated nasopharyngeal primaries. Besides, we noted that nasopharyngeal necrosis occurred relatively infrequently in our study (three out of 68 patients), which was a major contributing factor to severe epistaxis [40]. Notably, two patients experienced Grade 4 necrosis, both of whom had previously received high‐dose radiotherapy to the nasopharynx. On the background of radiation‐induced mucosal fibrosis, compromised vascular supply, and impaired tissue repair capacity, further inhibition of VEGFR/FGFR/PDGFR signaling by lucitanib may exacerbate local ischemia and vascular fragility, thereby predisposing to tissue breakdown and catastrophic bleeding in the irradiated field. Patients with high cumulative radiation doses, tumor extension to the skull base or major vessels, or severe preexisting mucosal damage should therefore be regarded as at increased risk. For such high‐risk patients, baseline and periodic nasopharyngoscopy or imaging, careful blood pressure control, avoidance of unnecessary anticoagulant/antiplatelet therapy or invasive procedures in the irradiated area, and prompt evaluation of recurrent epistaxis as a potential warning sign—with temporary interruption or dose reduction of lucitanib when appropriate and multidisciplinary management—may help to mitigate the risk of severe or fatal hemorrhage.

This study possesses certain limitations. Primarily, it employed a single‐arm design during the Phase II study. Despite its multicenter nature, a comparative analysis of the efficacy among immunotherapy monotherapy, chemo‐immunotherapy, and anti‐angio‐immunotherapy was absent. Hence, a randomized prospective trial is imperative to directly juxtapose each regimen. Secondly, the sample size within each tumor‐specific cohort remained modest and the enrolled population was heterogeneous, which constrains the generalizability of the efficacy estimates, particularly beyond NPC and EC. Further randomized controlled trials of this combination in larger, histology‐specific populations are warranted. In particular, for the EC cohort, MSI/MMR testing was not prospectively mandated and only a small subset of patients underwent heterogeneous local MSI/MMR assays with substantial missing data; therefore, the distribution of MSI/dMMR status could not be reliably determined and the observed activity should be interpreted as applying to an unselected population of recurrent or metastatic EC. In addition, we did not implement a preplanned, systematic biomarker program, and only limited, heterogeneous local tests were available, which precluded robust biomarker‐efficacy analyses and should be addressed in future studies. Finally, this analysis was based on a predefined data cut‐off of March 27, 2023, at which point investigator‐assessed ORR, the primary endpoint, and DCR were considered largely mature. In contrast, OS, DoR, and PFS were predefined as secondary, descriptive endpoints, and OS follow‐up is still ongoing. Accordingly, the current time‐to‐event estimates should be interpreted with caution and will benefit from longer‐term follow‐up in future analyses.

## 4 Conclusions

In summary, lucitanib combined with toripalimab showed promising antitumor efficacy and manageable safety profile in advanced solid tumors, with the most consistent benefit observed in immunotherapy‐treated NPC and recurrent EC. These findings, especially in these two tumor types, warrant further validation in larger, histology‐specific randomized controlled trials, while results in other tumor types should be considered exploratory.

## 5 Methods

### 5.1 Study Design and Participants

This multicenter, single‐arm, open‐label Phase II study was conducted at 19 study centers in China. The study protocol received approval from the Institutional Review Board of all participating institutions, and written informed consent was obtained from all participants. This study (AL3810‐101) comprised four parallel cohorts: (1) patients with recurrent or metastatic (R/M) NPC who had progressed after prior anti‐PD‐1/PD‐L1‐based therapy (immunotherapy‐treated NPC cohort); (2) patients with R/M NPC with no prior exposure to immune checkpoint inhibitors (immunotherapy‐naïve NPC cohort); (3) patients with recurrent or metastatic EC who had failed standard systemic therapy; and (4) patients with other advanced solid tumors that had progressed after, or were intolerant to, available standard treatments (“other tumors” cohort). Patients with histologically or cytologically confirmed advanced unresectable or metastatic solid tumors who failed at least one line of standard treatment were eligible for enrollment. Detailed inclusion and exclusion criteria are provided in the online supplemental methods. Major eligibility criteria included: (1) age between 18 and 75 years, (2) presence of at least one measurable lesion according to Response Evaluation Criteria in Solid Tumors V.1.1 (RECIST 1.1), (3) ECOG performance status score of 0–1, (4) life expectancy of at least 12 weeks, and (5) adequate organ function.

Key exclusion criteria were: (1) any Grade ≥ 3 hemorrhages within 4 weeks; (2) receipt of chemotherapy, immunotherapy, or targeted therapy within 4 weeks before study entry; (3) high risk of hemorrhage; and (4) active autoimmune diseases.

Exploratory biomarker assessments (such as PD‐L1 expression, MSI/TMB status, or immune profiling) were not mandated in the original protocol and were therefore only sporadically available from local routine clinical testing. Because of the heterogeneity in assay methods and the high proportion of missing data, no formal biomarker‐outcome analyses were performed in this Phase II study.

### 5.2 Procedures

Enrolled patients were administered lucitanib orally at a dosage of 10 mg once daily and toripalimab intravenously at a dose of 240 mg every 3 weeks for a duration of 2 years or until disease progression, death, intolerable toxicities, or withdrawal of consent. Dose modification of toripalimab was not allowed; however, dose interruption was permitted if Grade 3 or worse AEs occurred and were related to toripalimab. In cases where AEs were uncontrollable or deemed unacceptable, the dose of lucitanib could be reduced initially to 7.5 mg and subsequently to 5.0 mg, with no possibility of dose re‐escalation. If the interruption of lucitanib or toripalimab exceeded 2 weeks or 8 weeks, respectively, this led to permanent discontinuation of that drug in all cases. Specifics regarding dose reduction and interruption were outlined in the protocol.

### 5.3 Follow‐Up and Endpoints

Computed tomography scans or magnetic resonance images were conducted every two cycles (6 weeks ± 7 days) to evaluate tumor response according to RECIST 1.1 criteria. Clinical laboratory tests (complete blood count, serum biochemistry, coagulation profile, thyroid function, and urinalysis) were obtained at screening and at each protocol visit (Days 1, 8, and 15 of Cycles 1 and 3, Day 1 of Cycle 2, and Day 1 of each subsequent 21‐day cycle) until treatment discontinuation, and AEs were evaluated at every on‐treatment visit. After discontinuation, patients had a safety follow‐up visit 30 days after the last lucitanib dose or 60 days after the last toripalimab dose (whichever was later), and were then followed every 3 months for subsequent therapies and survival. AEs were graded using the National Cancer Institute‐Common Terminology Criteria for Adverse Events v5.0. AE monitoring occurred throughout the treatment cycles. The primary endpoint was the investigator‐assessed ORR, defined as the proportion of patients achieving complete response (CR) or partial response (PR) based on RECIST v1.1 criteria. Secondary endpoints included the DCR, defined as the proportion of patients with CR, PR, or SD; DoR, calculated as the time from the first documented objective response to disease progression or death; progression‐free survival (PFS), defined as the duration from treatment initiation to disease progression or death from any cause; and overall survival (OS), defined as the time from treatment initiation to death from any cause.

### 5.4 Statistical Analysis

The statistical analysis plan for this study was detailed in the protocol and in a predefined statistical analysis plan (SAP), employing Simon's minimax two‐stage design. In immunotherapy‐treated NPC cohort, the null hypothesis assumed an ORR of 20%, with a target ORR of 40% for the lucitanib plus toripalimab combination, at a one‐sided significance level of 10% and 80% power. Initially, 12 patients were enrolled, and if more than two responders were observed, enrollment would expand to 25 patients. The study outcome would be considered positive if more than seven patients responded to treatment, accounting for a 10% loss to follow‐up rate, with a planned inclusion of 28 patients in this cohort. A one‐sided α of 10% was chosen because this cohort was part of a single‐arm, signal‐seeking Phase II study primarily intended to screen for antitumor activity and to inform the need for subsequent confirmatory trials; in this early‐phase setting with a heavily pretreated and relatively rare population, a one‐sided α of 10% is commonly used in Simon's two‐stage designs to balance Type I error control against the feasibility of the required sample size.

For immunotherapy‐naïve NPC cohort, the null hypothesis assumed an ORR of 20%, while the alternative hypothesis posited an ORR of 50%, at a one‐sided significance level of 5% and 90% power. If two patients exhibited an objective response among the first 10 patients, enrollment would extend to 22 patients. The study would be deemed successful if more than seven responders were observed. Accounting for a 10% dropout rate, a total of 22 patients were enrolled in Cohort 2. For other tumor type cohorts, 30 patients were needed. For the EC and “other tumors” cohorts, which were planned as exploratory expansion cohorts, approximately 30 patients per cohort were intended (allowing for about 10%–15% non‐evaluable patients), without formal hypothesis testing. These sample sizes were chosen to provide reasonably precise descriptive estimates of ORR in each cohort and to allow detection of a potential efficacy signal if the observed ORR clearly exceeded the historical ORRs of approximately 10%–15% reported with standard therapies in similar settings.

The safety analysis and efficacy analysis were based on patients who received the lucitanib and toripalimab treatment regimen at least once; this population constituted both the full analysis set (for efficacy) and the safety analysis set. Baseline demographic and disease characteristics were summarized descriptively by tumor cohort. Continuous variables were presented as median (range) or mean (standard deviation), as appropriate, and categorical variables were presented as counts and percentages. Treatment‐emergent adverse events (TEAEs) were analyzed to assess safety. Tumor responses were assessed by investigators according to RECIST v1.1. The best overall response (BOR) was defined as the best tumor response recorded from the first dose until disease progression. For ORR and DCR, all patients in the full analysis set were included in the denominator; only patients with a BOR of CR or PR were considered responders for ORR, and patients with CR, PR, or SD (lasting ≥ 6 weeks) were considered responders for DCR. Patients without any post‐baseline tumor assessment or with only NE assessments were therefore included in the denominator but counted as non‐responders, consistent with an intention‐to‐treat approach. ORR, DCR, and their 95% CIs were calculated using the Clopper–Pearson method. The Kaplan–Meier method was used for the analysis of DoR, PFS, and OS, with survival curves plotted. Patients with no valid post‐baseline tumor assessment and no death were censored at the date of the first dose. Patients with at least one post‐baseline tumor assessment who had not experienced progression or death at the time of analysis were censored at the date of the last adequate tumor assessment (for PFS and DoR) or at the last date known to be alive (for OS). Patients who started a new systemic antitumor therapy before documented progression were censored at the date of the last adequate tumor assessment before initiation of the new therapy. Patients who withdrew consent or were lost to follow‐up without documented progression or death were likewise censored at the date of the last tumor assessment (for PFS and DoR) or at the last date known to be alive (for OS). For PFS and OS, all deaths, irrespective of their relationship to study treatment or underlying disease, were considered events, and potential competing events (such as deaths unrelated to treatment) were handled within these endpoint definitions; therefore, no separate competing‐risks model was applied. Efficacy and safety analyses by tumor cohort (including ORR, DCR, PFS, OS, and incidence of TEAEs) were prespecified in the protocol; additional subgroup and sensitivity analyses were exploratory and post hoc. Beyond the predefined Simon two‐stage designs in the NPC cohorts, all statistical analyses were descriptive and exploratory, and no adjustment for multiplicity was performed. All statistical analyses were conducted using SPSS 25 (IBM, Armonk, New York, USA).

## Author Contributions

Study concept and design: Li Zhang and Yunpeng Yang. Acquisition, analysis, or interpretation of data: Ting Zhou, Gang Chen, Yunpeng Yang, and Meng Li. Drafting of the manuscript: Ting Zhou, Haishuang Sun, Gang Chen, and Yunpeng Yang. Critical review of the manuscript for important intellectual content: Huiming Cai, Fugen Li, and Li Zhang. Statistical analysis: Ting Zhou, Yinbin Wang, and Meng Li. Obtained funding: Yunpeng Yang and Li Zhang. Administrative, technical, or material support: Guoping Zhang, Jinsheng Wu, Shenhong Qu, Yaqian Han, Desheng Hu, Yang Ling, Yulong Zheng, Jian Liu, Lizhu Lin, Yongsheng Li, Jianji Pan, Yanyan Liu, Cuiying Wang, Guohong Fu, Jian Feng, and Jianhua Shi. Supervision: Li Zhang and Yunpeng Yang. Li Zhang and Yunpeng Yang had full access to all the data in the study and take responsibility for the integrity of the data and the accuracy of the data analysis. All authors have read and approved the final manuscript.

## Funding Information

This study was supported by the Noncommunicable Chronic Diseases‐National Science and Technology Major Project (no. 2024ZD0519700, to Y.Y.), and the National Natural Science Foundation of China (nos. 82241232 and 82272789, to L.Z.; 82473203, to Y.Y.). Haihe Biopharma Co., Ltd funded this study and provided the lucitanib plus toripalimab used in it. The funding organizations had no role in the design and conduct of the study; collection, management, analysis, and interpretation of the data; preparation, review, or approval of the manuscript; and decision to submit the manuscript for publication.

## Ethics Statement

This study involves human participants and was approved by the ethics committees of all participating centers. Specifically, ethical approval was obtained from the ethics committee of Sun Yat‐sen University Cancer Center (approval number: A2021‐004‐01), Yuebei People's Hospital (approval number: Y‐2021‐12), The First Affiliated Hospital of Hainan Medical University (approval number: 2021 (Drugs) No. (12)), The People's Hospital of Guangxi Zhuang Autonomous Region (approval number: EC‐2021‐25(1)), Hunan Cancer Hospital (approval number: 2021 Drugs EC No. (282)), Hubei Cancer Hospital (approval number: (2021) No. 44), Changzhou Cancer Hospital (approval number: 2021‐SY‐023), The First Affiliated Hospital, Zhejiang University School of Medicine (approval number: 2021 EC No. (83)), The First Affiliated Hospital of Guangzhou University of Chinese Medicine (approval number: ZYYEC(2021)007), Chongqing University Cancer Hospital (approval number: CZLS2021088‐A), Fujian Cancer Hospital (approval number: 2021‐058‐02), Henan Cancer Hospital (approval number: 2021‐079‐001), Hainan Third People's Hospital (approval number: LL210379), Affiliated Hospital of Nantong University (approval number: 2021‐Y019‐01), and Linyi Cancer Hospital (approval number: XY2121). This trial was registered at the Chinese Clinical Trial Registry. Written informed consent was obtained from all patients before any study procedure, and the trial was conducted in accordance with the Declaration of Helsinki and Good Clinical Practice.

## Conflicts of Interest

Huiming Cai, Meng Li, Fugen Li, and Yinbin Wang are employees in Haihe Biopharma Co., Ltd. The other authors declare no conflicts of interest.

## Supporting information




**Supporting File 1**: Mco270672‐sup‐0001‐SuppMat.Docx

## Data Availability

All data generated or analyzed during this study are available from the corresponding author upon reasonable request.
