# Supplementary material for: Safety and Efficacy of Lucitanib Plus Toripalimab in Advanced Solid Tumors Refractory to Standard Therapies: An Open‐Label, Multicenter, Phase II Study
Source: MedComm (2020). 2026 Mar 11;7(3):e70672. doi: 10.1002/mco2.70672 (PMC13097439; doi:10.1002/mco2.70672)
Supplement: Supplementary file 1 — Supporting File 1: Mco270672‐sup‐0001‐SuppMat.Docx [file MCO2-7-e70672-s001.docx]

**Safety and efficacy of lucitanib plus toripalimab in advanced solid tumors refractory to standard therapies: an open-label, multicenter, phase II study**

**Running title:** Lucitanib Plus Toripalimab in Solid Tumors

Ting Zhou^1#^, Haishuang Sun^1^^#^, Gang Chen^1#^, Guoping Zhang^2^, Jinsheng Wu^3^, Shenhong Qu^4^, Yaqian Han^5^, Desheng Hu^6^, Yang Ling^7^, Yulong Zheng^8^, Jian Liu^9^, Lizhu Lin^10^, Yongsheng Li^11^, Jianji Pan^12^, Yanyan Liu^13^, Cuiying Wang^14^, Guohong Fu^15^, Jian Feng^16^, Jianhua Shi^17^, Huiming Cai^18^, Meng Li^18^, Fugen Li^18^, Yinbin Wang^18^, Li Zhang ^1^*, Yunpeng Yang ^1^*

1. Department of Medical Oncology, State Key Laboratory of Oncology in South China, Guangdong Key Laboratory of Nasopharyngeal Carcinoma Diagnosis and Therapy, Guangdong Provincial Clinical Research Center for Cancer, Sun Yat-sen University Cancer Center, Guangzhou 510060, China

2. Department of Medical Oncology, Yuebei People's Hospital, Shaoguan 512025, China

3. Department of Radiation Oncology, The First Affiliated Hospital of Hainan Medical University, Haikou 570102, China

4. Department of Otolaryngology Head and Neck, The People's Hospital of Guangxi Zhuang Autonomous Region, Nanning 530021, China

5. The second Department of head and neck Radiotherapy, Hunan Cancer Hospital, Changsha 410013, China

6. Department of radiotherapy, Hubei Cancer Hospital, Wuhan 430079, China

7. Department of Medical Oncology, Changzhou Cancer Hospital, Changzhou 213001, China

8. Department of Medical Oncology, The first affiliated hospital, Zhejiang university school of medicine, Hangzhou 310003, China

9. Phase I Clinical Trial Research Laboratory, The first affiliated hospital, Zhejiang university school of medicine, Hangzhou 310003, China

10. Department of Oncology, The first affiliated hospital of Guangzhou university of Chinese medicine, Guangzhou 510405, China

11. Department of Medical Oncology, Chongqing University Cancer Hospital, Chongqing 400030, China

12. Department of Radiotherapy, Fujian Cancer Hospital, Fuzhou 350014, China

13. Department of lymphatic integrative Medicine, Henan Cancer Hospital, Zhengzhou 450003, China

14. Department of Medical Oncology, Hainan Third People's Hospital, Sanya 572000, China

15. General Surgery Department, Hainan Third People's Hospital, Sanya 572000, China

16. Department of respiratory medicine, Affiliated Hospital of Nantong University, Nantong 226001, China

17. The second ward of the Department of Internal Medicine, Linyi Cancer Hospital, Linyi 276024, China

18. Haihe Biopharma Co., Ltd, Shanghai 201203, China

^#^Contributed equally to the study as co-first authors

*Contributed equally to the study as co-corresponding authors

***Corresponding Author:**

Yunpeng Yang, Department of Medical Oncology, Sun Yat-sen University Cancer Center, 651 Dongfeng Road East, Guangzhou, Guangdong 510060, P.R. China.

Tel: +86-20-87343368;

Fax: +86-20-87343368;

Email: [yangyp@sysucc.org.cn](mailto:yangyp@sysucc.org.cn)

Li Zhang, Department of Medical Oncology, Sun Yat-sen University Cancer Center, 651 Dongfeng Road East, Guangzhou, Guangdong 510060, P.R. China.

Tel: +86-20-87343458;

Fax: +86-20-87343535;

Email: zhangli@sysucc.org.cn

**Highlights**

- Lucitanib plus toripalimab showed efficacy in advanced solid tumors.
- The combination was active in PD-1-resistant NPC and recurrent EC.
- ORR reached 45.8% in immunotherapy-naïve nasopharyngeal carcinoma.
- Common grade ≥3 TRAEs included hypertension and proteinuria.
- Results support further study in PD-1-refractory NPC and EC.

**Supplementary Figures**

**Figure S1. Tumor response in patients with other solid tumors.** (A) Swimmer plot showing treatment duration and time to tumor response in patients with other advanced solid tumors. (B) Waterfall plot showing the best percentage change from baseline in target lesion size in patients with other advanced solid tumors. PR, partial response; SD, stable disease; PD, progressive disease; NE, not estimable.**
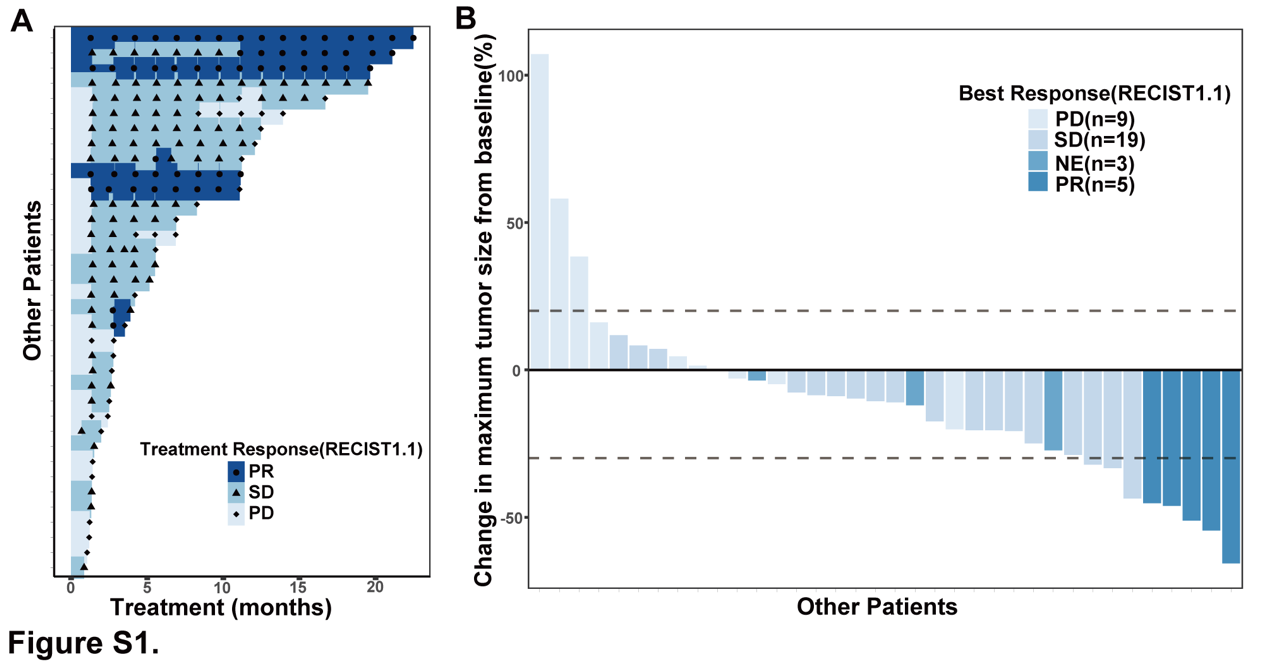
**

**Figure S2. Time-to-event outcomes in patients with other solid tumors.** (A) Kaplan–Meier curve of DoR in responding patients with other advanced solid tumors. (B) Kaplan–Meier curve of PFS in patients with other advanced solid tumors. (C) Kaplan–Meier curve of OS in patients with other advanced solid tumors. DoR, duration of response; NE, not estimable; PFS, progression-free survival.**
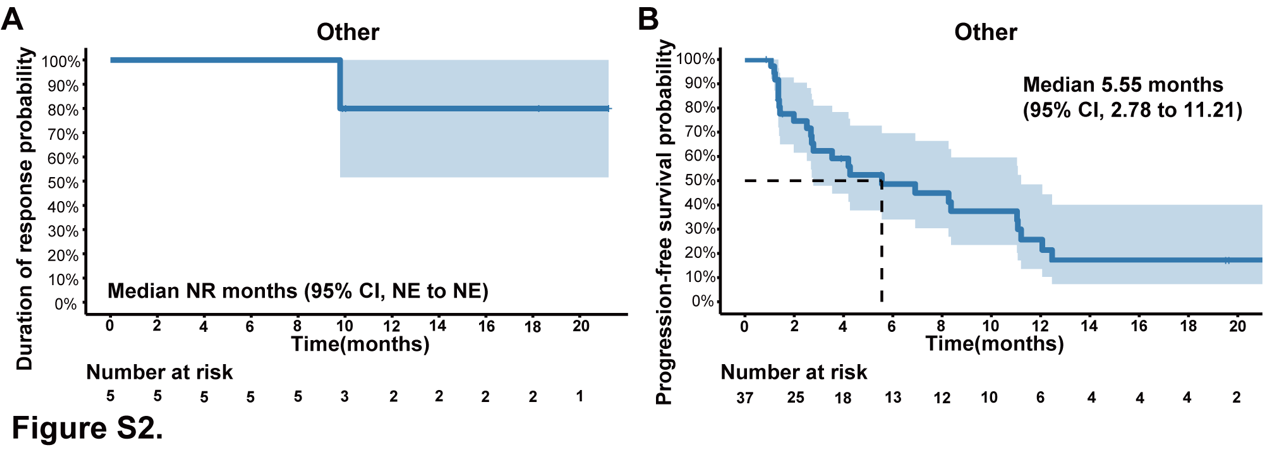
**
